# Exploring User Perceptions of a Mobile App for Religious Practices

**DOI:** 10.1007/s10943-024-02004-9

**Published:** 2024-02-15

**Authors:** Breanne Laird, Daryl R. Van Tongeren, Joshua N. Hook, Bridgette Do, Todd Hall, Jennifer Huberty

**Affiliations:** 1 Pray, Inc, 4607 Lakeview Canyon Rd #456, Westlake Village, CA 91361 USA; 2 Hope College, Holland, USA; 3 University of North Texas, Denton, USA; 4 University of Southern California, Los Angeles, USA; 5 Biola University, La Mirada, USA; 6 Fit Minded, Inc., Phoenix, USA

**Keywords:** Religion, Spirituality, Mental health, Digital health

## Abstract

The purpose of this study was to explore the usage patterns of USA subscribers of an online religious/spiritual application (i.e., app; Pray.com) and the associations of app usage with physical health, mental health, spiritual health, and well-being outcomes. A total of 1031 subscribers participated in the survey about their engagement with the Pray.com app. Most of the respondents had been using the app between one and two years, and more than half were high-frequency users. Although many individuals engaged with the app experienced spiritual growth, many also reported retrospective improvement in mental and physical health. This research serves as an initial examination of how religious-based apps may be associated with self-reported improvements in physical, mental, and spiritual health outcomes.

## Introduction

Technologically-mediated spiritual practices are becoming increasingly more common. The COVID-19 pandemic offered a glimpse into new ways by which individuals are engaging in religious and spiritual practices. Prior research has documented the associations between religious and spiritual practices with physical and mental health (Koenig, 2012; Paul Victor & Treschuk, 2020; Peteet, 2022). However, little is known about how practices that rely on technological media are similar to or differ from traditional religious and spiritual practices. The purpose of the current research was to begin to examine the perceptions of users of digitally mediated religious and spiritual practices.

Religion may be a boon for health. For example, religious individuals have a lower risk of mortality than non-religious individuals (Powell et al., 2003), and religion appears to lower risk for a wide range of physical health problems (Ellison & Levin, 1998). There also appears to be several benefits of religion for mental health; religious individuals often report higher levels of well-being and lower levels of mental health symptoms such as depression and anxiety (Peteet, 2022). Various mechanisms have been proposed for the religion-health link (Ellison & Levin, 1998), such as the regulation of individual lifestyles and behaviors (McCullough & Willoughby, 2009), social integration and support (Ellison & George, 1994), meaning in life (Steger & Frazier, 2005), and religious/spiritual coping (Pargament et al., 1998).

Approximately three out of four Americans report they identify with a specific religious faith, with Christians (69%) representing the largest percentage and approximately one in four Americans being Christians of color (26%) (Jones et al., 2021). The majority of Black Americans (79%) report they are affiliated with a religion and rely on prayer to help make major life decisions (Besheer et al., 2021). In 2020, almost 50% of adults belonged to a church, synagogue, or mosque (Jones, 2021). However, more recently, there has been a gradual and steady decline of individuals who identify as religious, especially as younger, less religious cohorts replace older and more religious populations (Packard & Ferguson, 2019). A Pew survey (2018) revealed that many individuals are now practicing their faith in “other ways”; seeking spiritual connections outside traditional religious structures (Center, 2018). For example, in 2020, the COVID-19 pandemic forced religious institutions to shut their doors to the public, which directly impacted the 36% of adults who attended religious institutions weekly (Pillay, 2020). As such, some in-person religious practices have shifted to digital formats, allowing individuals to continue their religious practice and sense of religious community online (Baker et al., 2020). Digital accessibility to religious practices may help mitigate daily obstacles, such as occupational and family demands, time, transportation, and overall cost of attending in-person religious services, allowing individuals to adapt to the demands of their lives while maintaining their religious/spiritual practices.

Although technology-mediated religious experiences may be increasing, research in this domain is lacking. Since 2020, religious and prayer-based applications (i.e., apps) have been burgeoning in the consumer market, increasing threefold since 2019 (Taylor, 2021). More people are turning towards digital media to facilitate their spiritual experiences, such as participating in chat groups with pastors, viewing online sermons, listening to religious podcasts, and engaging with religious content on social media (Melore, 2022). Modern technology is making organized religion much more appealing and accessible for millennials and younger generations (Melore, 2022). According to market research from PitchBook Data, venture capital funding for religious (primarily Christian-based) apps increased from $48.5 million in 2020 to $176.3 million in 2021 (Taylor, 2021), suggesting there is an increasing demand for app-based religious practices. Despite the growth in use of and investment in technology-mediated religious experiences, no studies have explored how users are engaging in these technologies and how they may be impacting health.

To examine our research question, we focused on one of the largest religious and prayer-based apps on the market. Pray.com is the world’s No. 1 app for daily prayer, with more than 12 million downloads. Content of the Pray.com app includes daily devotionals, Bible stories with original content, and Bedtime Bible stories narrated by inspirational speakers, faith leaders, or celebrities to help users sleep.

Given the increase of digitally-mediated spiritual practices and the lack of extant research in this area, the purpose of this study was to explore the usage patterns of United States (US) subscribers of an online religious/spiritual app (i.e., Pray.com) and the associations of app usage with physical health (i.e., overall physical health and sleep), mental health (i.e., overall mental health, stress, anxiety, depression, burnout), spiritual health (spiritual well-being, relationship with God, prayer life), and well-being (e.g., meaning and purpose in life) outcomes. Such findings will inform future interventions to test the effects of digital-based approaches for delivering religious/spiritual practices on mental health outcomes.

## Methods

### Ethics Approval

This study was approved by an Institutional Review Board at Biola University (STUDY F22-012). All participants provided electronic consent prior to participating in the survey. The deidentified datasets generated or analyzed during the study are available from the corresponding author upon request.

### Study Design/Recruitment

This study was cross-sectional. Participants were paying US subscribers to the mobile application, Pray.com, who were at least 18 years of age. Subscribers were recruited from October 2022 to November 2022.

### Participants and Procedure

Subscribers received an invitation to answer a series of quantitative questions related to their use of Pray.com and were informed that their answers would help improve their experience with the app and that results of this study may be used in reports, presentations, or publications. Potential participants were invited via push notifications, in-app messages, text messages, and emails. At the end of the survey, participants had the option of providing an email address to be entered into a drawing to win one of two $99 Amazon gift cards. No other identifying information was collected, and responses were not linked to the email provided or in-app usage data.

### Survey

The survey was developed by doctoral-level researchers in the field of psychology, behavior change, and religious/spiritual practices. The survey was administered online using Qualtrics and took most participants between 5 and 10 min to complete. All responses were kept separate from email addresses provided. Participants were asked to complete approximately 25 multiple-choice quantitative questions (not including demographics) about their engagement with the Pray.com app (e.g., frequency of use, reasons for using the app, content used, app features that support usage frequency) and whether they noticed improvements in health outcomes (e.g., physical health, mental health, stress, sleep, anxiety, depression, burnout, spiritual well-being, relationship with God, prayer life, and meaning and purpose in life) after using Pray.com. Questions varied slightly based on answers to the frequency of use. At the end of the survey, participants completed six questions about demographic characteristics.

### Statistical Analysis

Data were analyzed using IBM SPSS 27.0. For questions about demographic characteristics, reasons for starting to use Pray.com, the content of Pray.com used, app features that support usage frequency, and noticing health outcome improvements, participants were able to endorse multiple items from a list of options; selected items were treated as endorsements and unselected options were treated as non-endorsements. Chronic condition(s) were recoded as dichotomous (e.g., yes/no) and additionally recoded to reflect a chronic mental health condition (e.g., yes/no) and a chronic physical health condition (e.g., yes/no). Because respondents were able to endorse multiple chronic conditions, individual chronic condition(s) were recoded as dichotomous (e.g., yes/no). Because not all participants answered every question (i.e., survey was free response), sample sizes differed across analyses.

We report the data from all available completed data. Usage frequency was categorized as ordinal, reflecting use of the app or content of the app as (1) non-endorsed, (2) up to two times per week, (3) three to five times per week, or (4) five or more times per week. Variables for noticing improvements in health outcomes (i.e., physical health, mental health, stress, sleep, anxiety, depression, burnout, spiritual well-being, relationship with God, prayer life, and meaning and purpose in life) were ordinal reflecting: (1) no improvement, (2) *A little improvement*, (3) *Some improvement*, (4) *Moderate improvement*, (5) *Significant improvement*, and (6) *Extreme improvement*. Improvements in health outcomes were also recoded as dichotomous (i.e., significant to extreme improvement / non-endorsed to some improvement). Chi-square tests examined differences in self-reported dichotomous improvements in health outcomes based on frequency of app use and demographic characteristics (i.e., race, age, gender). In all cases, significant chi-square tests were followed up with *z*-tests of column proportions. *P* values were adjusted for multiple comparisons using Bonferroni correction. Because respondents were able to endorse multiple reasons for starting Pray.com and multiple types of content used and given the large number of answer choices for each of those questions, the relationships between reasons for starting and content used were described and detailed, but not statistically compared.

## Results

### Demographic Characteristics

A total of 1031 subscribers participated in the survey (see Table 1 for participation rates by invitation medium). The average age of respondents was 57 (*SD* = 12.42), with Gen X (i.e., 42–57 years of age; 35.31%, 64/1031) and Boomers (i.e., 58–67 years of age; 30.16%, 311/1031) being the most-represented age groups. Approximately two-thirds of respondents were female (68.87%, 710/1031). Most respondents identified as White (55.77%, 575/1031) and 26.29% identified as Black or African American (271/1031). Most respondents reported at least one chronic health diagnosis (62.85%, 648/1031) with physical health conditions more prevalent than mental health conditions (54.03%, 557/1031 vs. 35.21%, 363/1031). Sample demographics are presented in Table 2.

**Table 1:** Participation rates by invitation medium

Invitation type	Date range sent	Sent	Opened		Completed	
		N	n	%	n	%
Push notifications	10/26/22–11/01–22	339,026	4516	1.33	419	0.12
In-app messages	11/02/22–11/09/22	17,820	1832	10.28	331	1.86
SMS	11/10/22–11/12/22	7,630	7630	100.00	146	1.91
Email	11/16/22–11/16/22	196,356	1011	0.51	217	0.11
Total		560,832	14,989	26.73	1113	0.20

**Table 2:** Sample demographics (*N* = 1031)

Characteristic	n	%
Generation (*N* = 1015)		
Gen Z	14	1.38
Millennial	107	10.54
Gen X	364	35.86
Boomers	310	30.54
Older generations	220	21.67
Gender (*N* = 1015)		
Male	303	29.85
Female	710	69.95
Other	2	0.20
Prefer not to say	8	0.79
Ethnicity (*N* = 1031)		
Hispanic	73	7.08
Non-hispanic	958	92.92
Race (*N* = 1016)		
White/Caucasian	575	56.59
Asian/Asian American	7	0.69
Black/African American	271	26.67
American Indian/Native American or Alaska Native	10	0.98
Native Hawaiian or Pacific Islander	9	0.89
Biracial or multiracial	31	3.05
Other	40	3.94
Yearly Income (*N* = 811)		
$250,000 or more	67	8.26
$201,000–$250,000	41	5.06
$151,000–$200,000	75	9.25
$101,000–$150,000	153	18.87
$81,000–$100,000	111	13.69
$61,000–$80,000	115	14.18
$41,000–$60,000	145	17.88
Less than $20,000	104	12.82
Religious affiliation (*N* = 1022)		
Christian	939	91.88
Other	67	6.56
None	8	0.78
Latter-day saints	3	0.29
Muslim	2	0.20
Agnostic	2	0.20
Buddhist	1	0.10
Jewish	0	–
Hindu	0	–
Atheist	0	–
Chronic conditions (*N* = 1031)		
Anxiety	295	28.61
High blood pressure	291	28.23
Depression	273	26.48
Pain	186	18.04
High cholesterol	173	16.78
Arthritis or other rheumatic disease	170	16.49
Insomnia	136	13.19
Diabetes	122	11.83
Other	233	22.60
Asthma	82	7.95
Cancer	51	4.95
Heart disease	61	5.92
Emphysema or COPD	32	3.10
Other lung disease	18	1.75
None	367	35.60

### General App Usage Patterns

#### Length and Frequency of Use for Pray.com

Most respondents reported having used Pray.com for at least one year (59.94%, 618/1031; see Table 3). More than half of respondents were high-frequency users (i.e., more than five times per week; 58.78%, 606/1031) or moderate-frequency users (i.e., three to five times per week; 24.35%, 251/1031).

**Table 3:** App usage (*N* = 1031)

	n	%
Time used (*N* = 1030)		
More than 2 years	216	20.97
1–2 years	402	39.03
6–12 months	263	25.53
3–5 months	75	7.28
1–2 months	44	4.27
Less than 1 month	30	2.91
Frequency of use (*N* = 1025)		
More than 5 times/week	606	59.12
3–5 times/week	251	24.49
Less than 3 times/week	168	16.39
Reason for starting app		
Grow spiritually	905	87.78
Increase meaning and purpose	526	51.02
Reduce stress	480	46.56
Reduce anxiety	442	42.87
Overall health and well-being	418	40.54
Help in coping with crisis	354	34.34
Reduce depression	343	33.27
Improve sleep	323	31.33
Reduce burnout	138	13.39
Other	107	10.38
Curious	69	6.69
Friend recommended	53	5.14
Someone bought it for me	9	0.87

#### Reasons for Starting to Use Pray.com

The most common reason for starting to use Pray.com was to grow spiritually (87.78%, 905/1031; see Table 3). A substantial proportion of the sample also started using Pray.com because of the desire for improvements in mental and physical health as reasons for starting.

#### Content and Feature Use

The most widely used content on the app were Daily Prayer and bible reading. For example, Daily Prayer was the most popular content used (87.20%, 899/1031; see Table 4). Other popular content used by participants included Bible in a Year (46.75%, 482/1031), Bible Stories (42.39%, 437/1031), Bedtime Bible Stories (33.75%, 348/1031), and Meditative Prayers (32.88%, 339/1031).

**Table 4:** Self-reported app content and feature use

	n	%
App content used		
Daily prayer	899	87.20
Bible in a year	482	46.75
Bible stories	437	42.39
Bedtime bible stories	348	33.75
Meditative prayers	339	32.88
Reading the bible	310	30.07
Kids bible stories	204	19.79
Podcasts	194	18.82
21 day prayer journeys	147	14.26
Inspirational and celebrity voices	122	11.83
Worship music	122	11.83
Pray radio	109	10.57
PrayTV	36	3.49
App features that helped usage frequency		
Daily prayer reminder	538	52.18
None	359	34.82
Share streaks	188	18.23
Prayer journal	76	7.37
Pray.com community	70	6.79
App tracking	40	3.88
Other	23	2.04
Did not respond	12	1.16

Regarding features of the app that helped participants use the app more regularly, approximately 50% of respondents used Daily Prayer reminders to encourage them to use the app. Other features were used less often. Less than 20% of respondents used other in-app features that support usage frequency (e.g., Share Streaks, app tracking, Prayer Journal, Pray.com community). Approximately one-third of respondents had friends or family that used Pray.com, but utilization of the in-app community feature was low (6.79%; see Table 4). Of those with friends that used Pray.com, over 70% discussed the app among themselves.

### Improvements in Health Outcomes

Most respondents reported improvements in spiritual well-being outcomes [relationship with God (66.54%, 685/1031), prayer life (58.20%, 600/1031), and spiritual well-being (57.13%, 589/1031; see Table 4)]. Between 30 and 40% respondents reported improvements in overall mental health and stress, and well-being (i.e., meaning and purpose in life) while approximately 30% reported improvements in sleep and anxiety. Less than 20% of respondents reported improvements in physical health, depression, and burnout. Of those who reported improvements in their spiritual health, approximately 70% (see Table 5) considered their improvements in these areas to be “*Significant*”, or “*Extreme*”. Of those who reported improvements in mental health (overall mental health, stress, anxiety, depression, burnout), the majority considered their improvement to be “*Significant*”, or “*Extreme*”. Approximately 6% of respondents reported no improvements in any of the outcomes.

**Table 5:** Self-reported improvements in health outcomes

	n	%
Noticed improvement (N = 1031)		
Relationship with god	686	66.54
Prayer life	600	58.19
Overall spiritual well-being	589	57.13
Meaning and purpose in life	369	35.79
Overall mental health	405	39.28
Stress	357	34.63
Sleep	304	29.49
Anxiety	303	29.39
Depression	206	19.98
Overall physical health	122	11.83
Burnout	71	6.89
Other	41	3.98
Physical health outcomes		
Overall physical health (N = 122)		
A little improvement	5	4.10
Some improvement	11	9.02
Moderate improvement	34	27.87
Significant improvement	48	39.34
Extreme improvement	24	19.67
Sleep (N = 304)		
A little improvement	8	2.63
Some improvement	33	10.86
Moderate improvement	81	26.64
Significant improvement	127	41.78
Extreme improvement	55	18.09
Mental health outcomes		
Overall mental health (N = 405)		
A little improvement	6	1.48
Some improvement	47	11.60
Moderate improvement	99	24.44
Significant improvement	171	42.22
Extreme improvement	82	20.25
Stress (N = 357)		
A little improvement	9	2.52
Some improvement	50	14.01
Moderate improvement	107	29.97
Significant improvement	132	36.97
Extreme improvement	59	16.53
Anxiety (N = 303)		
A little improvement	5	1.65
Some improvement	44	14.52
Moderate improvement	88	29.04
Significant improvement	119	39.27
Extreme improvement	47	15.51
Depression (N = 206)		
A little improvement	8	3.88
Some improvement	31	15.05
Moderate improvement	54	26.21
Significant improvement	68	33.01
Extreme improvement	45	21.84
Burnout (N = 71) Burnout (N = 71)		
A little improvement	3	4.23
Some improvement	9	12.68
Moderate improvement	15	21.13
Significant improvement	27	38.03
Extreme improvement	17	23.94
Spiritual well-being outcomes		
Spiritual well-being (N = 589)		
A little improvement	7	1.19
Some improvement	33	5.60
Moderate improvement	140	23.77
Significant improvement	249	42.28
Extreme improvement	160	27.16
Relationship with god (N = 686)		
A little improvement	11	1.60
Some improvement	51	7.43
Moderate improvement	126	18.37
Significant improvement	284	41.40
Extreme improvement	214	31.20
Prayer life (N = 600)		
A little improvement	9	1.50
Some improvement	51	8.50
Moderate improvement	113	18.83
Significant improvement	216	20.95
Extreme improvement	211	31.17
Well-being outcomes		
Meaning and purpose in life (N = 369)		
A little improvement	4	1.08
Some improvement	20	5.42
Moderate improvement	70	18.97
Significant improvement	150	40.65
Extreme improvement	125	33.88

#### Differences Between Frequency of Use and Noticing Moderate to Extreme Improvements

There were several differences in self-reported improvement based on app usage frequency (see Table 6 for percent reporting improvement by group and omnibus comparisons).

**Table 6:** Frequency of use and noticing improvements in health outcomes

	Low (N = 168)		Moderate (N = 251)		High (N = 606)		df	χ2	*p*
	n	%	n	%	n	%			
Physical health	8	4.76	7	2.89	57	9.41	2	9.88	.007
Mental health	19	11.31	58	23.11	175	28.88	2	14.53	< .001
Stress	10	5.95	42	16.73	139	22.94	2	15.19	< .001
Sleep	22	13.10	41	16.33	119	19.64	2	1.20	.594
Anxiety	12	7.13	33	13.15	121	19.96	2	15.61	< .001
Depression	9	5.36	20	7.97	84	13.86	2	4.99	.083
Burnout	3	1.79	8	3.19	33	5.45	2	3.08	.214
Spiritual well-being	19	11.31	82	32.67	306	50.50	2	38.04	< .001
Relationship with god	38	22.62	93	37.05	365	60.23	2	29.95	< .001
Prayer life	26	15.48	74	29.48	325	53.63	2	33.03	< .001
Meaning and purpose in life	22	13.10	52	20.72	199	32.84	2	6.25	.044

#### Physical Health Outcomes

High-frequency users were significantly more likely than moderate-frequency users to report significant to extreme improvements in overall physical health (*χ*^*2*^_*2*=_ = 9.88, *p* = .007), but low-frequency users were not significant compared to high- or moderate-frequency users regarding noticing significant to extreme improvements in physical health.

#### Mental Health Outcomes

High-frequency users were more likely than low- and moderate-frequency users to report significant to extreme improvements in overall mental health (*χ*^*2*^_*2*=_ = 14.53, *p* < .001) and stress (*χ*^*2*^_*2*=_ = 15.19, *p* < .001); however, low- and moderate-frequency users did not significantly differ from each other. High-frequency users were more likely than low-frequency users to report significant to extreme improvements in anxiety (*χ*^*2*^_*2*=_ = 15.61, *p* < .001), but moderate-frequency users did not significantly differ from either group. Frequency of use was not related to the likelihood of reporting significant to extreme improvements in depression and burnout.

#### Spiritual-Well-Being

High-frequency users were more likely than low-frequency users to report significant to extreme improvements in spiritual well-being (*χ*^*2*^_*2*=_ = 38.04, *p* < .001), but moderate-frequency users were not significant compared to high- or low-frequency users regarding noticing significant to extreme improvements in spiritual well-being. High-frequency users were more likely than moderate- and low-frequency users to report significant to extreme improvements in their relationship with God (*χ*^*2*^_*2*=_ = 29.95, *p* < .001), and prayer life (*χ*^*2*^_*2*=_ = 33.03, *p* < .001).

#### Well-Being

High-frequency users were more likely than moderate-frequency users to report significant to extreme improvements in meaning and purpose in life (*χ*^*2*^_*2*=_ = 6.25, *p* = .044); but low-frequency users were not significant compared to high- or moderate-frequency users.

#### Differences Between Reasons for Starting Pray and Noticing Significant to Extreme Improvements

Regarding the most popular reasons for starting to use Pray.com, meaning and purpose was associated with improvements in overall physical and mental health, stress, spiritual well-being, relationship with God, and prayer life (see Table 7 for percent reporting improvement by group and omnibus comparisons). Using Pray to reduce stress was significantly associated with improvements in sleep, relationship with God, and prayer life (see Table 7 for percent reporting improvement by group and omnibus comparisons). To grow spiritually was not significantly associated with improvements in any health outcomes measured.

**Table 7:** Reasons for starting and noticing improvements in health outcomes

	Grow Spiritually (N = 905)		χ2	*p*	Increase Meaning and Purpose (N = 526)		χ2	*p*	Reduce Stress (N = 480)		χ2	*p*	df
	n	%			n	%			n	%			
Physical health	70	7.74	0.23	.629	58	11.03	5.89	.015	48	10.00	1.04	.307	1
Mental health	234	25.86	0.24	.621	175	33.27	6.83	.009	164	34.17	0.04	.851	1
Stress	177	19.56	0.12	.725	136	25.86	12.42	< .001	139	28.96	0.00	.973	1
Sleep	167	18.45	3.24	.072	102	19.39	0.75	.385	122	25.42	5.43	.020	1
Anxiety	153	16.91	1.08	.299	113	21.48	1.80	.180	120	25.00	0.05	.818	1
Depression	107	11.82	0.677	.411	73	13.88	0.13	.719	88	18.33	2.90	.089	1
Burnout	41	4.53	1.63	.202	34	6.46	2.68	.101	36	7.50	3.09	.079	1
Spiritual well-being	379	41.88	0.30	.581	253	48.10	2.11	< .001	200	41.67	1.20	.273	1
Relationship with god	462	51.05	1.12	.313	300	57.03	12.01	< .001	260	54.17	3.96	.047	1
Prayer life	395	43.65	0.01	.941	258	49.05	5.90	.015	220	45.83	8.16	.004	1
Meaning and purpose in life	258	28.51	0.28	.596	204	38.78	2.51	.113	164	34.17	2.02	.156	1

#### Differences Between Content Use and Noticing Significant to Extreme Improvements

Regarding the most popular content used, Daily Prayer was associated with the increased likelihood of reporting significant or extreme improvements in overall physical health, overall mental health, stress, anxiety, spiritual well-being, relationship with God, prayer life, and meaning and purpose in life. The use of Bible in a Year was associated with the increased likelihood of reporting significant or extreme improvements in overall mental health, anxiety, spiritual well-being, relationship with God, prayer life, and meaning and purpose in life. The use of Bible Stories was associated with the increased likelihood of reporting significant or extreme improvements in stress, sleep, anxiety, spiritual well-being, relationship with God, prayer life, and meaning and purpose in life (see Table 8 for percent reporting improvement by group and omnibus comparisons).

**Table 8:** Content used and noticing improvements in health outcomes

	Daily prayer (N = 899)		χ2	*p*	Bible in a year (N = 482)		χ2	*p*	Bible stories (N = 437)		χ2	*p*	df
	n	%			n	%			n	%			
Physical health	57	6.34	8.68	.003	33	6.85	0.38	.536	28	6.41	0.37	.544	1
Mental health	171	19.02	12.98	< .001	119	24.69	8.37	.004	101	23.11	3.83	.050	1
Stress	134	14.91	10.96	< .001	87	18.05	1.29	.256	82	18.76	4.50	.034	1
Sleep	109	12.12	0.24	.625	74	15.35	1.24	.266	92	21.05	10.28	.001	1
Anxiety	116	12.90	12.22	< .001	73	15.15	4.59	.032	69	15.79	4.02	.045	1
Depression	80	8.90	3.69	.055	51	10.58	1.12	.291	48	10.98	1.87	.172	1
Burnout	32	3.56	3.51	.061	20	4.15	0.13	.720	20	4.58	0.94	.332	1
Spiritual well-being	296	32.93	20.15	< .001	203	42.12	30.59	< .001	181	41.42	24.37	< .001	1
Relationship with god	351	39.04	26.08	< .001	237	49.17	29.20	< .001	219	50.11	37.49	< .001	1
Prayer life	316	35.15	28.12	< .001	200	41.49	30.60	< .001	175	40.05	16.31	< .001	1
Meaning and purpose in life	194	21.58	5.03	.025	137	28.42	4.71	.030	123	28.15	11.65	< .001	1

#### Differences Across Demographic Groups

##### Race

Because the two largest racial groups in our sample were White and Black participants, we tested for differences between these two groups. There were significant differences in the likelihood of reporting improvements in health outcomes, reasons for starting to use Pray.com, content used, and length of use across racial groups (see Table 9 for percent reporting improvement by group and omnibus comparisons). Black or African American respondents were more likely than White respondents to report moderate to extreme improvements in overall physical health (*χ*^*2*^_*1*=_ = 12.00, *p* < .001), overall mental health (*χ*^*2*^_*1*=_ = 11.59, *p* < .001), stress (*χ*^*2*^_*1*=_ = 10.21, *p* < .001), anxiety (*χ*^*2*^_*1*=_ = 5.94, *p* = .015), depression (*χ*^*2*^_*1*=_ = 7.45, *p* = .006), spiritual well-being (*χ*^*2*^_*1*=_ = 6.96, *p* = .008), prayer life (*χ*^*2*^_*1*=_ = 14.74, *p* < .001), and meaning and purpose in life (*χ*^*2*^_*1*=_ = 6.60, *p* = .010). Black or African American respondents were more likely than White respondents to report starting to use the app for help coping with a crisis (*χ*^*2*^_*1*=_ = 4.35, *p* = .037) and if a friend recommended the app (*χ*^*2*^_*1*=_ = 16.13, *p* < .001), and Black or African American respondents were more likely than White respondents to report using Meditative Prayer (*χ*^*2*^_*1*=_ = 5.70, *p* = .017) and Reading the Bible (*χ*^*2*^_*1*=_ = 8.23, *p* = .004). Black and African American respondents were more likely than White respondents to report having used the app for two or more years (*χ*^*2*^_*1*=_ = 18.35, *p* < .001), whereas White respondents were more likely than Black or African American respondents to have used the app for one to two years (*χ*^*2*^_*1*=_ = 9.98, *p* = .002). There were no significant differences across race with regard to frequency of use.

**Table 9:** Self-reported improvements by race

	Black & AA		White		χ2	*p*	df
	N = 271		N = 575				
	n	%	n	%			
Physical health	24	8.86	33	5.74	12.00	< .001	1
Mental health	115	42.44	84	14.61	11.59	< .001	1
Stress	84	30.10	65	11.30	10.21	< .001	1
Sleep	95	35.06	51	8.87	1.31	.253	1
Anxiety	88	32.47	47	8.17	5.94	.015	1
Depression	48	17.71	39	6.78	7.45	.006	1
Burnout	16	5.90	13	2.26	2.09	.149	1
Spiritual well-being	201	74.17	134	23.30	6.96	.008	1
Relationship with god	260	95.94	140	24.35	2.45	.117	1
Prayer life	192	70.85	152	26.43	14.74	< .001	1
Meaning and purpose in life	128	47.23	92	16.00	6.60	.010	1

##### Age Group

There were significant differences in the likelihood of reporting significant to extreme improvements in health outcomes across age groups (see Table 10 for percent reporting improvement by group and omnibus comparison). Gen X respondents (i.e., ages 42–57) were more likely than older respondents (i.e., 68–84 years of age) to report significant to extreme improvements in prayer life (*χ*^*2*^_*4*=_ = 19.76, *p* < .001). Other significant findings reported in Table 7 may be invalid due to small cell sizes (n < 5). There were no significant differences across age group with regard to frequency of use.

**Table 10:** Self-reported health improvements by age group

	Millennial		Gen X		Boomers		Older generations		df	χ2	*p*
	N = 107		N = 364		N = 310		N = 220				
	n	%	n	%	n	%	n	%			
Physical health	4	*3.75	30	8.24	22	7.10	12	5.45	4	11.19	.025
Mental health	35	32.71	99	27.20	70	22.58	45	20.45	4	3.13	.536
Stress	28	26.17	78	21.43	56	18.06	24	10.91	4	6.02	.198
Sleep	18	16.82	70	*19.23	56	18.07	31	14.09	4	5.16	.272
Anxiety	22	20.56	63	17.31	49	15.81	29	13.18	4	3.52	.476
Depression	20	18.69	51	14.01	27	8.71	13	5.91	4	7.69	.104
Burnout	3	2.80	30	8.24	7	2.26	3	1.36	4	7.20	.126
Spiritual well-being	43	40.19	158	43.41	121	39.03	80	36.36	4	1.88	.758
Relationship with god	53	49.53	184	50.55	148	47.74	101	45.91	4	3.57	.457
Prayer life	46	42.99	169	*46.43	125	40.32	74	33.64	4	19.76	< .001
Meaning and purpose in life	32	29.91	107	*29.40	83	26.77	49	22.27	4	9.68	.046

*Significant percentages across age groups

##### Gender

There were no significant differences in self-reported improvement or frequency of use across genders.

## Discussion

The purpose of this study was to explore technologically-mediated religious and spiritual practices. Namely, we examined the usage patterns of subscribers of a popular religious-based app (i.e., Pray.com), a mobile app designed to facilitate religious/spiritual practices, as well as the associations of usage with physical health, mental health, spiritual well-being, and well-being outcomes. This is one of the first investigations to explore usage patterns and associations with health outcomes among religious/spiritual mobile app users.

Most of the respondents had been using the app between one and two years, and more than half were high-frequency users (i.e., more than five times per week). Although almost all the app subscribers reported that they used the app to grow spiritually, many participants also reported that they started to use the app because of mental and physical health concerns. For example, almost half used the app to reduce stress and one-third used the app to reduce anxiety. Regarding the content most used by participants, daily prayer was the most popular component of the app.

The majority of respondents reported improvements in their relationship with God, over one-third reported improvements in their overall mental health and stress, and almost one-third reported improvements in their sleep and anxiety. Reporting improvements in overall mental health and stress was more likely in high- and moderate-frequency users as compared to low-frequency users. There were differences in self-reported improvements in health outcomes based on frequency of app use and race and age.

Most respondents reported being long-term (i.e., using the app for more than one year) and moderate to high-frequency users (i.e., using the app at least three times per week). The most common reason for starting to use the app was to grow spiritually and many participants also started to use the app for mental and physical health benefits. Consistent with this purpose of using the app, many participants noticed improvements in overall physical and mental health. Given the prior research on the salutary effects of religion on health (Ellison & Levin, 1998; Koenig, 2012; Powell et al., 2003), it is unsurprising that individuals would turn toward mobile apps designed to facilitate religious involvement as a way to maintain their religious practice when and where it was convenient (Bellar, 2017) and also to enhance their physical and mental health. Because prior work on technology-mediated religious activities and health-related outcomes is largely nascent, this work contributes to this growing area.

Daily Prayer was the most popular app content used and approximately half of respondents used the in-app feature, Daily Prayer reminders, to support usage frequency. Many respondents had friends who also used Pray.com, but usage of the in-app community feature was low. Our findings suggest the majority of users engage in prayer privately as a way to enhance their health and well-being, despite the conflicting (if not limited) scientific evidence that doing so has health benefits (Breslin & Lewis, 2008; Masters & Spielmans, 2007). Interestingly, although social support is one of the key mechanisms that has explained the link between religious involvement and health benefits (George et al., 2009), it appears that engagement in the mobile app (at least in our sample) is primarily a private activity. Future research is warranted to explore ways to engage practitioners with in-app communities and the impact that this might have on using the app and the health benefits associated.

Almost half of the respondents used the app to reduce stress and one-third to reduce anxiety. Over one-third reported improvements in their overall mental health and stress, whereas almost one-third noticed improvements in their sleep and anxiety. In general, respondents reported noticing improvements in the domain that brought them to the Pray.com app. For example, almost half of those who used the app for stress reported improvements in stress. It is not surprising that users are turning to a religious app to help them with their stress and anxiety, as belief systems play a beneficial role in coping with stressful experiences, especially those that are difficult to explain (Koenig, 2018; Pargament et al., 2000). It is also not surprising that of those who used the app to reduce stress, almost half reported improvements in overall mental health and stress. A study by McCullough et al. suggests those who are affiliated with a religion and have high levels of religious involvement are at reduced risk for depressive symptoms and disorders (McCullough & Larson, 1999). These findings support that technology-mediated religious experiences, such as Pray.com, are associated with increased self-reported mental health improvements and symptom-specific improvements. More research is needed to explore the effects that engagement with religiously-based mobile apps has on mental health.

When examining self-reported improvements, we found that greater improvements in overall mental health and stress were reported among high and moderate-frequency users compared to low-frequency users. High-frequency users were more likely to report improvements in several domains as compared to lower-frequency users. This suggests a possible dosage effect in self-reported improvement, such that greater use of religious technology is related to increased self-reported improvement. These findings warrant the need to better understand the behavior of low-frequency users and explore ways to increase engagement. Future research could test in-app education on the “ideal” usage for potential improvement in health outcomes or explore various motivations in high-frequency vs. low-frequency users.

We also explored the relationship between demographic characteristics (i.e., gender, age, race) and frequency of using the app but found no significant associations between age, gender, or race and frequency of use. Although there were no significant demographic differences in app usage, there were some significant demographic differences regarding health improvements from using the app. With regard to race, Black or African American respondents were more likely than White respondents to report improvements in overall physical health, overall mental health, stress, anxiety, depression, spiritual well-being, prayer life, and meaning and purpose in life. This is consistent with previous research that has found that African Americans have a more elaborate prayer life, including higher rates of praying for guidance for one’s health (Krause & Chatters, 2005). In addition, Chatters et al. (2008) found African Americans were more likely to endorse the importance of prayer when dealing with stress and more likely to look to God for strength, support, and guidance compared to non-Hispanic Whites. Prior research has also suggested higher levels of religious participation among African Americans (Taylor et al., 1996), as well as the strong influences of religion/spirituality in African American culture (Johnson et al., 2005). Previous literature posits that Black individuals may be more likely to reap the health-related benefits of religion because they are more involved in it (Krause, 2002). For example, Black respondents who attended church more frequently reported a greater decline in somatic depressive symptoms, whereas church attendance was not associated with somatic depressive symptoms among White respondents (Krause, 2003). Further research is needed to understand why Black or African American respondents were more likely than White respondents to report improvements in overall physical and mental health, stress, anxiety, depression, spiritual well-being, prayer life, and meaning and purpose in life after participating in digital religious practices regardless of frequency of use.

With regard to age, there was a significant association between generational age groups and self-reported health outcomes. Gen X respondents (i.e., ages 42–57) were more likely than older respondents (i.e., 68–84 years of age) to report significant to extreme improvements in prayer life (*χ*^*2*^_*4*=_ = 19.76, *p* < .001). This may be due to Gen X having more experience/comfort using mobile apps. Gen X didn’t use the app more, but their experience/comfort may have contributed to improvements in prayer life as their comfort with technology is more comparable to that of millennials than older generations (Calvo-Porral & Pesqueira-Sanchez, 2020).

Finally, we explored the relationship between reasons for using the app and self-reported improvements in health outcomes, as well as the relationship between content use and health outcomes. Previous research has demonstrated that religion is a primary pathway for meaning in life. Specifically, prior studies have found that meaning is the conduit through which religion enhances well-being (Chamberlain & Zika, 1992; Ivtzan et al., 2013; Steger & Frazier, 2005). Consistent with this, participants reporting using the app to increase meaning and purpose was associated with improvements in overall physical and mental health, stress, spiritual well-being, relationship with God, and prayer life. This suggests that motivations to enhance meaning as associated with flourishing and spiritual engagement. When participants reported using the app to reduce stress, it was significantly associated with improvements in sleep, relationship with God, and prayer life. Indeed, such associations are consistent with prior research demonstrating the role of religion in mitigating stress (Pargament & Park, 2019; Park, 2005) and its associations with health (Graham et al., 2001; Siegel et al., 2007). Interestingly, indicating the use of the app to grow spiritually was not significantly associated with improvements in any health outcomes measured. Given previous research documenting associations between spirituality and mental health (Hall, 2004), we encourage future research to explore this relationship further.

Of the top three most popular content used, the use of Daily Prayer was significantly associated with improvements in overall physical health, overall mental health, stress, anxiety, spiritual well-being, relationship with God, prayer life, and meaning and purpose in life. Although the cross-sectional nature of this study precludes causal statements, it is possible that regular engagement in a daily activity is associated with greater self-reported flourishing and well-being. Certainly, greater religious involvement (Ellison, 1991) and religious rituals or habits (Sohi et al., 2018) have been linked with well-being. Similarly, the use of Bible in a Year was significantly associated with improvements in overall mental health, anxiety, spiritual well-being, relationship with God, prayer life, and meaning and purpose in life, and the use of Bible Stories was associated with significant improvements in stress, sleep, anxiety, spiritual well-being, relationship with God, prayer life, and meaning and purpose in life. Although there are myriad reasons why individuals may seek out particular content, and cross-sectional designs are limited in their explanatory power, these associations should be further explored in longitudinal designs; moreover, respondents were able to endorse multiple reasons for starting to use Pray.com and content used, so future feasibility studies should also be conducted.

### Limitations

This was a descriptive, survey-based study and there are a number of important limitations to consider. First, the sample was a convenience sample of Pray.com users who used the app quite frequently, had been using the app for quite some time, and most respondents were female and identified as White. Additionally, the survey did not include those who used the app less than up to two times per week, decreasing participation rates of infrequent users. Future research should explore perceptions in more diverse samples in terms of gender, race/ethnicity, and pattern of usage. Second, the survey was designed specifically for this study by investigators. Because of space constraints, the survey was brief and consisted of mostly single-item measures. Future studies could extend the findings of this paper by using validated measures and questionnaires to assess mental health. Third, with regard to Pray.com content use, many respondents used Daily Prayer, Bible in a Year, and Bible Stories, and it is difficult to parse out the unique effects of these components. Future research could randomize participants to use one specific religious content to assess the benefits of specific content on mental health. This information could be presented alongside qualitative data to inform future experimental studies. Fourth, the retrospective ratings of self-improvement are subject to bias. For example, people may overreport improvement in areas where there may have objectively been little change (Jayawickreme et al., 2021). Future research should employ longitudinal designs to assess these dimensions over time, rather than relying on retrospective self-reports. Fifth, the investigators did not ask follow-up questions for respondents that did not see improvements. Although the percentage of people who did not report an improvement in the outcomes measured was low, future research should ask follow-up questions to better understand if no improvement was observed or if symptoms or perceptions of the outcome may have worsened. Sixth, the investigators did not ask questions regarding if respondents attend in-person religious practice or are involved in a religious community outside of the Pray.com app. Participation in a religious community outside Pray.com participation may have impacted the findings and future work should explore this possibility. Lastly, a cross-sectional, nonexperimental study design yields no evidence for causation or direction. We cannot infer causality regarding the impact of Pray.com as the analyses relied on subscribers’ self-reported recollection of app usage, perceptions of improvement in various health outcomes, and their beliefs about how Pray.com has impacted them. Future studies should explore these relationships prospectively, and randomized clinical trials are needed to determine the extent to which improvements in health outcomes can be attributed to Pray.com usage.

## Conclusion

Individuals are becoming increasingly exposed to technology-mediated religious activities. However, previous research has not explored this new frontier of religion and spirituality. This is the first study to explore the usage patterns of a sample of paid subscribers of a religious/spiritual mobile app (i.e., Pray.com) and the associations of usage with self-reported health outcomes as well as the associations of self-reported health outcomes with regard to user demographics. Although many individuals engaged with the app to experience spiritual growth, many also reported retrospective improvement in mental and physical health. Despite some methodological limitations, this research serves as an initial examination of how religious-based apps may be associated with self-reported improvements in physical, mental, and spiritual health outcomes. This study lays the foundation for future clinical trials and longitudinal studies aimed at determining the efficacy of religious-based apps in improving health outcomes.
